# Editorial: Symbioses Between Protists and Bacteria/Archaea

**DOI:** 10.3389/fmicb.2021.709184

**Published:** 2021-07-29

**Authors:** Alexei Yu. Kostygov, João M. P. Alves, Vyacheslav Yurchenko

**Affiliations:** ^1^Faculty of Science, Life Science Research Centre, University of Ostrava, Ostrava, Czechia; ^2^Zoological Institute of the Russian Academy of Sciences, St. Petersburg, Russia; ^3^Department of Parasitology, University of São Paulo, São Paulo, Brazil; ^4^Martsinovsky Institute of Medical Parasitology, Sechenov University, Moscow, Russia

**Keywords:** protists, prokaryotic symbionts, metabolism, ecology, genomics, holobiont concept

Symbiosis is a regular long-term cohabitation of organisms of two (or more) species, of which at least one partner benefits. Such relationships can be classified as parasitism, commensalism, or mutualism, depending on whether they are harmful, neutral or beneficial to another partner (Douglas, 2010). The evolution of eukaryotes was always driven by various symbioses with prokaryotes. The very origin of this group about 1.5 billion years ago was associated with the acquisition by a proto-eukaryote of an α-proteobacterial endosymbiont, which later became the mitochondrion. The rise of algae and, later, land plants, which significantly changed the whole biosphere, became possible owing to the establishment of endosymbiotic relationships with cyanobacteria, which turned into plastids (Keeling et al., 2015).

Symbiotic associations with prokaryotes were most intensively studied in plants and animals because of their practical importance, whereas much less attention has been paid to those in protists. Meanwhile, studying this phenomenon in unicellular eukaryotes is advantageous, since they represent simpler and at the same time more diverse models, allowing its deeper investigation. The studies presented in this Research Topic were focused on the diversity, stability, and specificity of symbiotic associations between protists and bacteria/archaea, as well as symbionts' genomics, metabolic contribution and their role in shaping the relationships of parasitic protists with animals.

Some protists may host entire consortia of microorganisms, as exemplified by the giant amoeba *Pelomyxa palustris* processing sapropel with the help of one archaeal and two bacterial endosymbionts (Gutiérrez et al., 2017), or the termite gut-inhabiting parabasalian *Trichonympha* spp., combining bacterial endo- and ectosymbionts in order to feed on wood particles (Stephens and Gage, 2020). These relationships may range from transient facultative associations, such as those between *Acanthamoeba* spp. and various bacteria (Guimaraes et al., 2016), to finely-tuned (as a result of a long-term coevolution) obligate systems observed in trypanosomatids of the subfamily Strigomonadinae (Alves et al., 2013; Silva et al., 2018).

For many symbiotic associations the exact nature of relationships between protists and their prokaryotic cohabitants is uncertain. However, there are examples of apparent mutualism, e.g., *Pandoraea novymonadis* in the trypanosomatid *Novymonas esmeraldas* (Kostygov et al., 2017) or *Phycorickettsia trachydisci* in the eustigmatophyte alga *Trachydiscus minutus* (Yurchenko et al., 2018) and parasitism e.g., *Holospora* spp. in *Paramecium* spp. (Fokin and Görtz, 2009).

Some symbioses are medically relevant, when a protist carries bacteria pathogenic to humans, e.g., *Mycoplasma hominis* in *Trichomonas vaginalis* (both obligately parasitic in genital tract) or *Legionella pneumophila, Vibrio cholera, Listeria monocytogenes, Escherichia coli*, etc. in facultatively parasitic acanthamoebae. Such associations were reviewed in this Research Topic by Henriquez et al., who demonstrated that the presence of bacterial symbionts may significantly aggravate the pathogenesis of infections by *T. vaginalis* and *Acanthamoeba* spp.

Hunter et al. analyzed the genomes and transcriptomes of the apicomplexan *Cardiosporidium cionae* and its yet unnamed bacterial endosymbiont of the family Rickettsiaceae. This parasite and the related mutualistic genus *Nephromyces* inhabit tunicates and represent the only Apicomplexa carrying symbiotic bacteria. The genomic analysis revealed that *C. cionae* obtains essential nutrients, including lysine and lipoic acid, from its endosymbiont. The authors propose that the metabolic contribution, provided by three endosymbionts of *Nephromyces*, allowed it to become mutualistic to its tunicate host, whereas the contribution of a single bacterium of *Cardiosporidium* is not sufficient for such a transition.

The work of Weiler et al. investigated resistance of *Paramecium caudatum* to the endonuclear parasitic bacterium *Holospora undulata* in different strains isolated in numerous countries across the world. Although the resistance varied significantly, the authors detected its positive correlation with genetic and geographic distances between the ciliate strains. For example, *P. caudatum* from Europe appeared to be more susceptible to the infection by European bacteria. This suggests that the farther away the populations of *P. caudatum* are from each other, the higher are the chances that their defense mechanisms differ and the strategy used by *H. undulata* would have to be changed.

Mironov and Sabaneyeva investigated another endosymbiotic association including *Paramecium*, but a different species, *P. multimicronucleatum*. Similarly to the case of *Cardiosporidium cionae* mentioned above, it hosts a bacterium of the family Rickettsiaceae, namely *Ca*. Trichorickettsia mobilis. In contrast to the *Paramecium*-*Holospora* system, the relationship between the ciliate and its intranuclear symbiont is robust and the latter cannot be removed by any of the four antibiotics tested (ampicillin, streptomycin, chloramphenicol, tetracycline), each with a different mode of action. The antibiotics either did not kill any of the partners of this symbiotic association or, at high concentrations, caused the death of both. As the authors demonstrated, tetracycline, the drug most efficient against bacteria of the family Rickettsiaceae, is also toxic to the ciliates. This has also been previously shown for several other protist groups. Ampicillin and chloramphenicol caused the formation of dormant persisters out of active *Ca*. Trichorickettsia mobilis cells, contributing to the stability of the system under study. Based on that, the authors proposed this symbiotic association as a model to elaborate the concept of the holobiont (an organism with its associated microbiota), originally introduced by Husnik et al. (2021).

The topic of symbiosis in such fascinating and diverse group as protists can hardly be exhausted and surely has many aspects still unclarified or completely unknown. As new lineages of protists are discovered every year, the vast amount of their diversity still remains to be explored. There is a rather limited number of endosymbiotic systems involving protists established as experimental models amenable to genetic modification. Such models would allow detailed investigation of the mechanisms underlying the complex relationships between the eukaryotic and prokaryotic partners. With the new cellular, molecular and genomic technologies and approaches applied to the study of symbiosis in protists, the pace of discovery in the field is sure to increase in ever accelerating rate. After the acceptance of this paper for publication, a relevant review on the topic has been published (Margulis, 1991). We address the readers to it for further information about the symbioses between protists and bacteria and/or archaea.

## Author Contributions

All authors listed have made a substantial, direct and intellectual contribution to the work, and approved it for publication.

## Conflict of Interest

The authors declare that the research was conducted in the absence of any commercial or financial relationships that could be construed as a potential conflict of interest.

## Publisher's Note

All claims expressed in this article are solely those of the authors and do not necessarily represent those of their affiliated organizations, or those of the publisher, the editors and the reviewers. Any product that may be evaluated in this article, or claim that may be made by its manufacturer, is not guaranteed or endorsed by the publisher.
